# Complete Genome Sequences of Nine Mycobacteriophages from New Zealand, Beatrix, Carthage, Daegal, Dulcie, Fancypants, Fenn, Inca, Naira, and Robyn

**DOI:** 10.1128/MRA.00395-19

**Published:** 2019-05-16

**Authors:** C. G. Davies, J. K. Wojtus, C. E. R. Gilligan, S. F. Latu, T. J. Manning, C. E. Meyer, J. Nersesyan, R. I. Tennent, L. Oosthuizen, E. Palmer, A. F. M. Hoskin, J. Turnbull, J. A. Sweatman, N. Elliston-Boyes, C. J. Wallace, A. Baxter, Z. Borrie, J. S. Elder, O. Massey, A. Wong, O. K. Silander, N. E. Freed, H. L. Hendrickson

**Affiliations:** a School of Natural and Computational Sciences, Massey Phage Whānau, Massey University, Auckland, New Zealand; b College of Medical, Veterinary & Life Sciences, University of Glasgow, Glasgow, United Kingdom; Queens College

## Abstract

Beatrix, Carthage, Daegal, Dulcie, Fancypants, Fenn, Inca, Naira, and Robyn are newly isolated bacteriophages capable of infecting Mycolicibacterium smegmatis mc^2^ 155. We discovered, sequenced, and annotated these New Zealand bacteriophages. These phages illustrate that New Zealand harbors a selection of the highly diverse and distributed mycobacteriophage clusters found globally.

## Abstract (precis)

Beatrix, Carthage, Daegal, Dulcie, Fancypants, Fenn, Inca, Naira, and Robyn are newly isolated bacteriophages capable of infecting Mycolicibacterium smegmatis mc^2^ 155. We discovered, sequenced, and annotated these New Zealand bacteriophages.

## ANNOUNCEMENT

The bacteriophages of mycobacteria have been deeply sampled as a result of the intensive discovery and sequencing efforts of the Howard Hughes Medical Institute’s Science Education Alliance–Phage Hunters Advancing Genomics and Evolutionary Science (SEA-PHAGES) program (http://phagesdb.org) (1, 2). Among these is mycobacteriophage StepMih (GenBank accession number MF185733), the first sequenced from New Zealand (3). Here, we report on the discovery, purification, sequencing, and cluster designations of the following nine new mycobacteriophages: Beatrix, Carthage, Daegal, Dulcie, Fancypants, Fenn, Inca, Naira, and Robyn. These bacteriophages infect the host strain Mycolicibacterium smegmatis mc^2^ 155 and were isolated from around Auckland, New Zealand, as part of the SEA-PHAGES program. This varied set of bacteriophages from an isolated island can help us to understand the relationship between geography and diversity in bacteriophages (4).

Phages Inca and Fenn were isolated from rural agricultural locations; Fenn was found in soil on an abandoned metal door, and Inca was found in soil under a silage bale. Beatrix, Naira, Carthage, and Daegal were found in soil and compost from the Massey University garden. Phages Dulcie and Fancypants were isolated from compost on urban properties, and phage Robyn was discovered in soil beneath grass clippings.

Phage Inca was obtained using the enrichment method. Briefly, M. smegmatis mc^2^ 155 was propagated in 7H9 liquid medium supplemented with albumin, dextrose, and 1 mM CaCl_2_. This were added to a compost sample, filtered using a 0.22-μm filter, and incubated for 96 hours with M. smegmatis mc^2^ 155 at 37°C. The direct plating method was used to isolate the remaining eight bacteriophages. Briefly, phage buffer (10 mM Tris [pH 7.5], 10 mM MgSO_4_, and 68 mM NaCl) plus 1 mM CaCl_2_ was added to soil or compost samples. These were mixed well and filtered using a 0.22-μm filter. After filtration, all samples were assayed for plaque formation on M. smegmatis mc^2^ 155 by double agar overlay with host M. smegmatis mc^2^ 155. All bacteriophages were plaque purified and amplified to produce high-titer lysates. DNA was extracted from Inca using the Promega Wizard DNA cleanup kit (Promega catalog number A1120). DNA was extracted from the remaining eight phages using the zinc chloride precipitation method (5). Sequencing libraries were constructed using an NEB Ultra II FS kit with dual-indexed barcoding. Libraries were pooled and sequenced on an Illumina MiSeq instrument to produce single-end 150-base reads with a minimum of 245,473 reads (see Table 1). Raw reads were submitted to Newbler v.2.9 (with default settings) for assembly and yielded single-phage contigs in all cases. Contigs were checked for completeness, accuracy, and phage genomic termini using Consed v.29 as previously described (6). The finalized genomes had a minimum 522-fold coverage (Table 1). All nine bacteriophages were assigned to clusters based on a minimum 50% alignment to previously sequenced mycobacteriophages according to a BLASTN sequence similarity analysis (Table 1) (6).

**TABLE 1:** Mycobacteriophage genome details organized by cluster^*a*^

Phage name	Cluster	Genome length (bp)	G+C content (%)	Fold coverage (×) (no. of reads)	No. of tRNAs	No. of CDS^*b*^	GenBank accession no.
Beatrix	A1	52,295	63.9	2,101 (777,138)	0	90	MK305893
Dulcie	A1	53,970	63.7	522 (346,235)	0	94	MK494116
Fenn	A1	53,449	63.7	754 (533,235)	1	97	MK310141
Naira	A1	53,581	63.7	649 (245,473)	1	95	MK524523
Carthage	B1	67,952	66.6	1,461 (703,425)	0	101	MK524512
Robyn	B1	68,467	66.6	1,096 (531,596)	0	102	MK524526
Inca	E	74,070	62.8	2,299 (1,229,065)	2	147	MH576956
Fancypants	F1	60,228	61.1	1,574 (660,308)	0	110	MK524503
Daegal	Q	54,858	67.5	3,483 (1,344,677)	0	87	MK494095

a GPS coordinates can be found at http://phagesdb.org (1).

b CDS, coding sequences.

Genome annotations were performed using DNA Master, which integrates BLAST, GeneMark, and Glimmer (http://cobamide2.bio.pitt.edu/), PECAAN, which integrates BLAST and HHPRED (https://pecaan.kbrinsgd.org/), and Phamerator (6).

Electron microscopy was conducted at the University of Auckland Electron Microscopy facility. All nine bacteriophages belong to the family *Siphoviridae* and have isometric capsids (see images at http://phagesdb.org).

### Data availability.

The genome sequences reported here have been deposited in GenBank under the accession numbers provided in Table 1. Raw data are available at BioProject number PRJNA488469.
